# Exercise for People with Parkinson’s Disease: Updates and Future Considerations

**DOI:** 10.1298/ptr.R0030

**Published:** 2024-07-19

**Authors:** Jennifer L. MCGINLEY, Yasuhide NAKAYAMA

**Affiliations:** ^1^Physiotherapy Department, Melbourne School of Health Sciences, The University of Melbourne, Australia; ^2^Department of Rehabilitation Medicine, The Jikei University School of Medicine, Japan

**Keywords:** Parkinson’s disease, Exercise, Physiotherapy

## Abstract

Parkinson’s disease (PD) is now the world’s fastest-growing neurological disorder with rapidly rising prevalence and increasing demand for effective health services. Recent research has focused on the importance of early diagnosis and proactive management of physical function. Accumulating evidence indicates that reduced physical activity levels and mild pre-clinical disability are present in many people prior to a clinical diagnosis, perhaps developing over years. Early referral to a physiotherapist at the time of diagnosis is now recommended in global guidelines. Multiple forms of exercise have been found to have benefits in early and mid-stage disease across a range of motor and non-motor symptoms. Evidence from longitudinal studies confirms that disability is delayed when regular exercise is sustained over long periods. Exercise is now recognized as an essential component of treatment, in combination with medical therapies. Contemporary physiotherapy interventions now combine health behavior change techniques with physical exercise to promote the development of long-term exercise adherence. Advances in technology and digital health have progressed quickly and now offer opportunities for remote assessment and monitoring, remote exercise supervision, and support adherence through feedback and motivational strategies. Recent biomedical discoveries forecast improved earlier and more accurate diagnosis of PD, allowing opportunities for earlier interventions. Current research in progress will provide important insights into the dose and intensity of aerobic exercise in PD. Physiotherapists have important roles in advocacy and education in conjunction with care delivery to support access to evidence-based care for all people with PD.

## Introduction

Parkinson’s disease (PD) is a common progressive neurological disease thought to affect around 9 million people by 2030^1)^. The World Health Organization recognizes PD as the fastest-growing neurological disorder in terms of disability, hence health policy and healthcare planning must consider the increasing burden on healthcare systems^1)^. Estimates of the number of people with PD in Japan vary, ranging from 0.136^2)^ to 0.26 million^3)^. The social and economic burden is substantial and increases with disease progression, with annual care costs per person estimated to be 225,929 JPY in early disease and 250,025 JPY in advanced disease^4)^. As the population in Japan continues to age, with an increasing prevalence of age-related neurological conditions, effective management of people with PD is of key importance. Although treatments for PD continue to develop, there is no cure, and symptom management across the lifespan is essential. Progression is inevitable, although the rate of deterioration varies considerably and many people live long lives with PD, even decades^5)^. Physiotherapy plays a key role in multidisciplinary teams as an adjunct to medical interventions, enabling people to maintain their optimum function and quality of life.

Characterized by the loss of dopamine-producing cells in the basal ganglia, PD causes both motor and non-motor symptoms that affect a person’s ability to perform activities in everyday life and restrict engagement and participation in social and community life. Common motor features include movement slowness, tremors, rigidity, postural instability, and difficulty with balance and gait^5)^. Walking is often slow with small steps and freezing of gait is associated with an increased risk of falls^6)^. Non-motor symptoms have been less well recognized but are equally common and include fatigue, sleep disturbance, mood changes including depression or anxiety, cognitive impairment, and autonomic dysfunction^5)^. Both motor and non-motor symptoms vary in presence and severity and may fluctuate with medication cycles (on/off phenomenon). Recent accumulating evidence also indicates that women experience PD differently from men, with lesser motor progression and a different profile of non-motor symptoms including greater mood and sleep disturbances, fatigue, and pain^7)^. This complex interaction of symptoms leads to increasing difficulty with activities of daily living including mobility, communication, social interaction, and employment. Motor progression is typically characterized by Hoehn and Yahr staging (see Table 1), representing progression from unilateral disease to bilateral, with trunk and balance involvement and increasing difficulty with walking, progressing to non-ambulant status in advanced disease^8)^. The complex combination of both motor and non-motor symptoms in PD underscores the importance of physiotherapy to address the diverse needs of individuals living with this chronic condition.

**Table 1.** Hoehn and Yahr stages

Stage	Hoehn and Yahr Scale
1	Unilateral involvement only usually with minimal or no functional disability
2	Bilateral or midline involvement without impairment of balance
3	Bilateral disease: mild to moderate disability with impaired postural reflexes; physically independent
4	Severely disabling disease; still able to walk or stand unassisted
5	Confinement to bed or wheelchair unless aided

From Goetz et al.^8)^

Physiotherapists are recognized as experts in exercise and physical activity interventions. This paper aims to provide an overview of recent evidence from this field and highlights new areas of interest and future opportunities.

## Exercise for People with PD is Recommended to Address Motor and Non-Motor Symptoms

The World Health Organization, National Institute of Clinical Evidence (UK), and other guideline authors now emphasize the need for access to expert integrated multidisciplinary services for people living with PD^1^^,^^9^^–^^12)^. An International and Multidisciplinary Rehabilitation Medicine Task Force recently developed a comprehensive consensus statement regarding multidisciplinary rehabilitation in PD care^9)^. Optimal physiotherapy was considered important across all stages of the disease, from diagnosis to advanced PD. Physiotherapy-specific guidelines are now emerging, including those from Europe and the United States^11^^,^^13)^. Medical rehabilitation guidelines in Japan support the key role of physiotherapy for the management of PD as a member of a multidisciplinary team, recognizing exercise as effective and safe^14)^. Motor rehabilitation is recognized as effective in improving physical function, quality of life, gait speed, and fall risk reduction^15)^. Detailed physiotherapy-specific guidelines from the Japanese Physical Therapy Association also recommend exercise from diagnosis onwards, providing specific recommendations across stages including a range of exercise modalities, acknowledging the variable strength of evidence and knowledge gaps^16)^.

Positive evidence to support the benefits of physiotherapy and exercise in the routine care of people with PD has increased over the last decade, in both volume and quality. Progress has been rapid; the first Cochrane Review of Physiotherapy for people with PD in 2013 included only 39 randomized controlled trials (RCT), with only 8 trials identified as low risk of bias^17)^. A recent Cochrane review of physical exercise in 2023 identified 154 RCTs, with generally higher quality methodology, supporting the beneficial effects of many types of exercise on a range of motor outcomes and quality of life^18)^. The effective modalities that positively influence motor outcomes include (but are not restricted to) dance, aqua therapy, gait/balance/functional programs, mind–body, strength/resistance, and endurance programs^18)^. The effective modalities that positively influence the quality of life include (but are not restricted to) aqua therapy, endurance activity, mind–body, gait/balance/functional programs, and strength exercises^18)^. Importantly, the interventions were considered relatively safe, with no adverse effects^18)^. The volume of evidence from recent meta-analyses addressing specific symptoms or functional difficulties is growing rapidly, confirming exercise as effective in improving freezing of gait^19)^, hand dexterity^20)^, and falls^21)^, a significant burden for many people with PD.

As the body of evidence has grown, systematic reviews with meta-analyses have sought to compare the relative benefits of different types of exercise modalities and to understand whether certain forms of exercise are superior or best. As the contributing studies are usually heterogeneous across participants, intervention dose, and duration, with relatively few direct evaluations, network meta-analyses (NMA) provide the most evidence through the evaluation of direct and indirect effects. Broadly, these results confirm relatively small differences between exercise types and confirm that multiple modalities are effective^18^^,^^22^^,^^23)^. As the methodology varies across reviews and exercise modalities are classified into different types, the results differ substantially across studies. One large NMA has examined specific modalities in greater detail, finding, for example, positive benefits from power training for motor symptoms and body weight supported treadmill training on balance and gait speed and distance^23)^. The consolidation of positive findings from systematic reviews that address many symptoms is encouraging; however, caution is needed to interpret the evidence carefully and apply it to individual patients due to the large variation in the effect sizes and strength of evidence, treatment modalities, and outcomes assessed. Physiotherapists must consider the person’s presenting symptoms, their goals, the presence of any comorbid health conditions, access, and preferences, and identify relevant evidence to support shared decision-making conversations in treatment decisions^24)^.

As awareness of the significant burden of non-motor symptoms has increased over the last decade, attention has further focused on the potential effect of exercise and regular physical activity on these symptoms. It is also now recognized that some non-motor symptoms may present as barriers to regular exercise, such as apathy, depression, fatigue, or cognitive impairment. A systematic review evaluating the associations between physical inactivity and sedentary behavior confirmed that higher sedentary time is associated with worse cognition, with longitudinal cohort studies also suggesting low activity levels were associated with more depression and impaired cognition^25)^. This highlights the importance of physiotherapists understanding and assessing both the motor and non-motor symptoms of PD and considering the impact on exercise adherence and treatment choices.

Systematic reviews are now exploring the effect of physical exercise on non-motor symptoms. A recent large umbrella review including 20 reviews of physical activity or exercise interventions confirmed that exergame, dance, resistance exercise, aerobic exercise, and combined interventions promoted improved cognition, although the optimal dose and duration remain uncertain^26)^. This effect on cognition was confirmed in a separate review^27)^, with a further suggestion that the effect was primarily on executive function, rather than other aspects of cognition. A smaller review focused on people with early to mid-stage PD (Hoehn and Yahr (H&Y) mean <2.5) found low-quality evidence that tango and mixed treadmill training improved non-motor symptoms, assessed using the MDS-UPDRS Part 1^22)^. Exercise has also been found in meta-analyses to have a moderate to large effect on depression and a smaller on anxiety^28)^.

## Early Intervention: Diagnosis and Early-Stage Physiotherapy

The appropriate timing of rehabilitation interventions for people with PD has received considerable attention over recent years. Traditionally, referral to physiotherapy and other disciplines was considered appropriate when disability emerged, or symptoms interfered with function. The expectation was that therapy would provide compensatory approaches to adapt to inevitable motor decline rather than directly seek improvement in function through motor learning, skill acquisition, and exercise or seek to actively delay disability progression. In parallel, there has been increasing recognition that a PD diagnosis typically occurs after many years of preclinical disease progression and that preclinical changes in function may have already occurred. For example, around 25% of people with very mild disease (UPDRS < 20; median H&Y < 2) reported changes in walking, including slowness or greater difficulty^29)^. A detailed review of subtle motor deficits prior to diagnosis included changes in gait patterns, balance, facial movement, and finger tapping, emerging between 2 and 10 years prior to clinical diagnosis^30)^.

Contemporary recommendations now indicate that referral to physiotherapy or exercise health professionals is recommended at the time of diagnosis. This aligns with the adoption of an early and active ‘secondary prevention’ model, rather than a delayed rehabilitation program^31)^. Late referrals, therefore, represent a “lost opportunity” to introduce pro-active and preventative measures in early disease to reduce worsening of disability or inactivity^31)^. Early referral may provide opportunities for education regarding the benefits of sufficient regular physical activity and provide direction to community-based exercise programs. Baseline assessment using standardized measures and person-centered goal-oriented measures allows for regular monitoring over time, addressing motor function, fall risk, and home or community exercise programs in the early stages of PD^9)^. As almost one in four people with PD are typically diagnosed while working age (< 65 years)^5)^, particular attention to motor and non-motor demands of the person’s occupational role is warranted. Knowledge from the field of occupational health should be used in conjunction with PD-specific evidence to ensure that people remain employed for as long as they choose and if possible. A recent large survey from the European Parkinson’s Disease Association across 28 European countries examined the impact of employment on PD^32)^. Symptoms that contributed to individuals leaving employment included fatigue, mobility, manual dexterity, sleep, and other non-motor symptoms^32)^. Less than one in four people had received early intervention to manage their condition and considered this may have helped them remain at work. This study, in conjunction with others, suggests that physiotherapy may have a key role in supporting employment in people with PD who are working at the time of diagnosis.

Notably, around the time of PD diagnosis, a decline in daily activity and reports of increased difficulty with walking are common. A review of incident (newly diagnosed) cohorts with PD confirms that many people have lower physical activity levels than their peers. When diagnosed, the ICICLE cohort was around one-third less than peers without PD, taking fewer daily steps, and walking for fewer long (>2 minutes) bouts^33)^. Similarly, loss of postural control may emerge earlier in PD than previously thought. In a group of newly diagnosed individuals with PD, around one in four had falls prior to diagnosis, and around one in three then fell within 18 months of diagnosis^34)^. Given the evidence revealing the prevalence of inactivity early in the course of PD and the known benefits of exercise, it seems early initiation of physiotherapy has the potential to have the most impact^35)^.

Isolating the effect of exercise on people at diagnosis or in very early disease is challenging, as most studies have included heterogeneous populations. Recent studies have focused on the efficacy of interventions for people with early disease, such as those who are de novo and taking no medication. A small RCT of inpatient multidisciplinary intensive rehabilitation treatments (MIRT) of 3 hours daily, 5 days a week for 4 weeks evaluated patients across 2 years. Compared to a control (no MIRT) group, the MIRT group had better motor scores, activities of daily living (ADL) scores, timed up and go, and lesser disability, indicating lesser disability progression^36)^. Similarly, the SPARX RCT recruited de novo participants to participate in a trial of high and low-intensity treadmill exercise relative to usual care, finding that the higher-intensity program was feasible and had positive changes in the UDPRS motor scores, relative to the control group^37)^. Participants with low initial step counts (<4200 daily steps) in the high-intensity treadmill exercise group also showed a large significant increase in daily step count and minutes of moderate-to-vigorous physical activity^38)^.

## Sustaining Long-Term Exercise Habits Over Long Periods: Health Behavior Change Support is Needed

Current frameworks suggest that people with PD should maintain or commence regular exercise from diagnosis onwards and develop lifelong exercise habits. Most of the evidence from RCTs has however evaluated only short-term benefits and trials extending beyond 2 years are rare. Given that PD progresses slowly over many years, useful insights into longer-term benefits can be gained from cohort studies.

Li and colleagues^39)^ followed a cohort of 143 people with PD who trained in Tai Chi twice weekly over 4+ years. Compared to a control group, the Tai Chi group progressed less slowly annually (UPDRS total score), had smaller medication increases, maintained better mobility and balance, and had fewer complications^39)^. Similar findings were evident in a large cohort of 237 people with early PD in Japan, followed over 5 years^40)^. Higher physical activity levels over time were associated with a slower decline in postural and gait stability, ADL, and processing speed^40)^. Similar findings were suggested in a single cohort of 55 people with PD who exercised over 5 years; annual progression of disease (1.7%) was considered lower than typical changes in general PD samples^41)^. Collectively, these studies support the ongoing benefits of sustained regular exercise for people with PD over extended periods. Other studies also indicate the benefit of commencing regular exercise after previously being inactive; a 30-minute increase in exercise per week was associated with small positive changes in health-related quality of life and mobility^42)^.

Sustaining enough exercise at regular intervals over long periods can be challenging. Like other chronic conditions, research has begun to focus on the self-management skills of people with PD, seeking factors that support long-term lifestyle modification including habitual exercise behavior. Multiple studies have now identified factors that may be modifiable and hence potentially useful in physiotherapy programs. Ellis et al.^43)^ used the International Classification of Functioning and Disability framework to explore exercise behavior determinants in a group of 260 people with PD categorized as regular exercisers or non-exercisers. Exercise self-efficacy was found to be a key factor; people with high exercise self-efficacy were twice as likely to exercise than those with lower self-efficacy^43)^. Exercise self-efficacy was defined as “a person’s belief in his or her capabilities to overcome personal, social, and environmental barriers to exercising”^43)^. Older age and higher education were also significant factors associated with exercise. Higher disease severity was associated with exercise in initial univariate analysis but did not remain significant in multivariate analysis. Importantly, this study highlighted that the disabling influences of impairments and activity and participation limitations and restrictions were not associated with exercise behavior^43)^. Further factors associated as negative influencers of exercise have included low-outcome expectations, lack of time, fear of falling, poor perceived health, bad weather, and lack of an exercise partner^44^^,^^45)^. Notably, enjoyment of exercise was identified as a positive predictor of exercise^45)^.

The presence of carer support has been identified as a potential facilitator of regular exercise behavior. A recent study evaluated the habitual physical activity of newly diagnosed people with PD with carers over an 18-month period^46)^. Although activity levels did not change over time, carer psychosocial factors were associated with the physical activity levels of their PD partner; lower levels of carer anxiety and depression were associated with higher activity volume, and worse carer self-care was associated with a greater loss of physical activity volume^46)^. This suggests that physiotherapists could consider including carers within interventions to improve physical activity and support and further consider the emotional well-being of the dyadic unit^46)^.

Recognition of the challenge of long-term exercise adherence and identification of personal factors associated with exercise has led to the integration of health behavior change methods in physiotherapy interventions to increase exercise volume and adherence. For example, the large ParkFIT RCT integrated a behavior change program as an adjunct to an exercise program, including an activity coach who used strategies including education, barrier identification, goal setting and contracts, and feedback on activity levels^47)^. Other studies have used peer coaches, virtual coaches, and mobile health apps for monitoring and telephone support or telehealth^48^^–^^52)^. The theoretical frameworks underpinning these interventions have varied across trials, including social cognitive theory, Bandura’s self-efficacy theory, self-determination theory, the health belief model, and the trans-theoretical (stages of change) model among others^53)^. Most interventions have been multi-component and there is no clear evidence that one specific behavioral approach is better than another^53)^. Contemporary guidelines now recognize the importance of integrating health behavior change methods into interventions to increase or sustain exercise over the lifespan. Many trials are now also focused on home and community-based exercises, such as walking or stationary cycles, which address barriers such as access, transport, and time restrictions and facilitate recruitment of more diverse participant cohorts^54)^.

## Advances in Technology and Digital Health

Rapid changes to the capability, cost, and availability of technology in healthcare have provided opportunities for physiotherapy care for people with PD. Technology can support multiple aspects of care, enhancing assessment, as an integrated component of physical or behavior change interventions, and for remote monitoring of physical activity or exercise performance to provide patient feedback or tailored remote exercise progression.

Wearable sensors worn in daily life and during home exercise programs allow important insights into “real-world data.” Sensors worn on the lower back over a week by 587 people with PD found the data to be moderately associated with clinical measures, and sensitive to early disease stage and disease progression^55)^. Sensors integrated into commercially available devices such as the Parkinson’s Kinetigraph (PKG^TM^) are now used in neurologist-led care, to evaluate patients and optimize medication^56)^. As PD typically includes movement variations and fluctuations within and across days, these insights are particularly important. Data such as these provide insights into on and off times and dyskinesia and may thus also allow physiotherapists to provide personalized education to identify optimum times for exercise and community activity and to consider periods of elevated fall risk. Such sensors are now being evaluated as a key component of the new home-based healthcare pathway model in the UK which combines supported self-management, remote monitoring, and self-directed access to healthcare contacts if needed^57)^. Early results suggest that the model is acceptable, feasible, and safe and may be useful for remote or underserved communities^57)^.

Sensors in body-worn devices have also been used in conjunction with specialized health software to provide opportunities to personalize exercise reminders, deliver encouragement, and feedback through messages, and tailor exercise progression remotely^51)^. They do however have limitations including uncertain accuracy, cost, access to the sensors, hardware placement issues, and ease of data retrieval and interpretation. The effect of custom sensors to increase the physical activity of people with PD appears promising, yet requires further exploration^58)^.

As an alternative to wearable sensors, existing integrated sensors within smartphones also offer opportunities for remote assessment, due to the widespread uptake of smartphones globally and the everyday habits of carrying phones. Various studies have explored data collected in everyday activity and in defined movement protocols to be used in home testing. The large mPower study captured data over 6 months from over 12,000 individuals (1414 with PD) who completed defined motor tasks or activities up to three times daily: finger tapping, speaking, walking, balance, and memory^59)^. Performance in the motor tasks in a small subset of 21 people with PD found a reasonable correlation with objective clinical measures of disease severity (UPDRS_MDS total score)^59)^.

The wide consumer access to smartphones also offers new opportunities to develop and evaluate interventions that may be able to be implemented in remote areas or larger populations where healthcare services are limited. The STEPWISE RCT (in progress^60)^) aims to increase physical activity in people with PD, using a remote app (STEPWISE); integrating motivational elements (virtual coach, positive text messages, personalized feedback about step count targets) and remote step count monitoring. The app is automated, thus testing the feasibility of an intervention that does not require human supervision. Remote assessment via smartphone offers exciting opportunities for personalized monitoring in routine physiotherapy practice yet will require careful consideration of the accuracy and the limitations of interpretation, and the acceptability of digital interventions to the person.

The development of augmented and virtual reality systems (AR and VR) has also provided new opportunities to address motor impairments, such as in gait or balance training. Virtual reality technology simulates the real world or similar environments, enabling a user to interact with a 2D or 3D environment using movement. It may be immersive (e.g., wearable headset) or non-immersive (computer screen or game console interface) and may include custom-developed or commercially available games (“exergaming”). A key advantage of VR is the ability to provide a safer physical environment while allowing the person to attempt challenging movement tasks.

Virtual reality as a component of physiotherapy for PD has been evaluated using both customized programs developed for research and commercially available systems. Furthermore, the use of such systems provided attractive opportunities during the COVID-19 pandemic. A recent systematic review confirmed the efficacy of VR in improving balance and balance confidence, with no significant change in gait, ADL, or quality of life^61)^. VR may also provide opportunities to combine aerobic exercise with a dual task or exergaming component. For example, the V-TIME trial added VR to treadmill exercise, requiring participants to navigate a simulated obstacle course with cognitive challenges, with real-time visual feedback of walking^55)^. The VR-enhanced treadmill intervention was more effective in reducing fall risk than a treadmill without a VR. Individual studies have varied widely, with marked diversity across the types and demands of VR, the supervision required, and the dose. This confirms the potential advantages but indicates that additional knowledge is needed to identify the optimal dose and protocol, degree of challenge, and longer-term effects^61)^. Commercially available systems offer obvious advantages in terms of access, cost, and availability, and emerging evidence suggests promising findings. A recent review of the effect of Nintendi Wii^TM^ and Xbox Kinect^TM^ on gait in PD suggested that both systems showed positive effects, with a small NMA providing limited support for Wii as a potentially superior device to influence gait^62)^.

## Future Directions: Improved Diagnostic Certainty and the Potential of Exercise as Neuroprotection

Emerging biological data are now also driving efforts to better define the diagnosis of PD and classification of phenotypes and staging, which is important for prognostic and therapeutic studies. Currently, the diagnosis is primarily clinical with limited accuracy and occurs late in the disease process, with around 60% of dopaminergic nigrostriatal neurons lost prior to diagnosis^63)^. Diagnostic errors are common, with around 20% of people misdiagnosed at initial assessment^64)^. The recent discovery of a new biomarker (α-synuclein seed amplification assays) in the well-characterized Parkinson’s Progression Markers Initiative Cohort is anticipated to lead to biomarker-defined-at-risk cohorts and improve diagnostic certainty of PD for participants in new therapeutic trials^65)^. In addition, the potential for wearable sensor data to support the inclusion of movement data to identify PD prior to clinical diagnosis is also emerging. Using the UK BioBank data, researchers used machine learning models trained using accelerometry data to distinguish between people with clinically diagnosed PD, Prodromal-PD, and healthy controls up to 7 years pre-diagnosis^66)^. Over the next decade, research trials will likely benefit from improved definitions, which will amplify efforts in disease modification trials, including those including exercise.

The use of biomarkers to improve understanding of the effect of exercise and physical activity on disease progression in people with early PD has also expanded over the last 5 years. Extending from rodent models of PD that indicate neuroprotective effects on dopaminergic neurons, exercise studies are now integrating measures of brain-derived neurotrophic factor (BDNF) and imaging as outcome measures. A recent systematic review evaluated the effect of exercise on outcomes related to PD disease progression, finding limited low-certainty evidence showing improvement in “off” UPDRS scores and BDNF concentration^39)^.

Although exercise and physical activity are now universally recommended for PD, much uncertainty remains as to the appropriate mode, frequency, and intensity that is necessary. Multiple exercise forms provide benefits and experts suggest multifaceted programs including elements such as aerobic, strengthening, motor-cognitive challenges (such as agility/balance/dual task, etc.), and flexibility. Aerobic exercise and the targeted intensity of exercise are particularly interesting in the studies exploring potential neuroprotective effects. Positive findings of the recent SPARX2 study^37)^ and Park-in-Shape trial^67)^, which both included aerobic exercise at moderate-high intensity, provide strong support for recommendations that people with PD engage in aerobic exercise 3×/week, 30- to 40-minute main exercise set, 60%–80% of heart rate reserve or 70%–85% of heart rate max^68)^. In lieu of heart rate, individuals can achieve an intensity of 14–17 on a 20-point rated perceived exertion (RPE) scale^68)^. Current trials in progress that are nearing completion will provide a greater understanding of the appropriate dose for early PD (H&Y 1-III). These include the SPARX3 trial^69)^ (NCT04284436) which will evaluate 18 months of moderate and high-intensity endurance exercise for people within 3 years of diagnosis who are de-novo. Similarly, the CYCLE-II study (NCT04000360) evaluates a home-based high-intensity aerobic program on a stationary cycle over 12 months^54)^.

Two recent key studies have provided important and exciting insights into the neural mechanisms underlying the positive effect of aerobic exercise on motor symptom progression^70^^,^^71)^. De Laat and colleagues evaluated 10 people with mild disease (H&Y = 2, mean disease duration of 2 years) who engaged in high-intensity exercise over 6 months and found an improved or stable motor function^70)^. Notably, positron emitting tomography imaging of dopamine transporter availability in the striatum and substantia nigra found a significant increase, rather than the expected decrease. Neuromelanin sensitive MRI identified similar changes in neuromelanin concentration in the substantia nigra; an increase rather than a decrease^35)^. Similarly, a small study comparing functional and structural magnetic resonance imaging from people in the Park-in-Shape trial found enhanced functional connectivity in the right frontoparietal network and reduced global brain atrophy in those engaged in aerobic exercise, but not in the stretching group^71)^. Together, these studies suggest aerobic exercise improves functionality in the remaining dopaminergic neurons and alters brain structure and function^70^^,^^71)^.

The association between physical activity and PD is also now increasingly recognized in epidemiological studies examining the risk of developing PD. A very large systematic review including more than half a million participants found that physical activity was associated with a significant reduction in risk of PD^72)^. Higher levels of physical activity and exercise were associated with a lower risk of PD, but notably, this occurred only in men. Related to the physical activity-PD risk focus, new research directions are considering the potential for regular exercise as part of a multicomponent lifestyle PD program^73)^. An overview of current trials in progress that address physical activity in PD highlights seven RCTs expected to be completed over the next 2 years^74)^. All of these RCTs focus on strategies to increase the overall volume of physical activity in everyday life, all are home-based, and all include a remote intervention component^74)^. Knowledge from these trials should provide important information about adherence to regular exercise as part of lifestyle change.

The phases of PD development and progression are also the focus of considerable research interest. A prodromal phase (prodromal-PD) of 10–15 years prior to clinical diagnosis has been identified, characterized by non-motor symptoms including hyposmia, constipation, REM sleep behavior disorder, and mood disorders^5)^. A first-of-its-kind trial (Slow-SPEED-NL NCT06193252) has just commenced, to assess the possible efficacy of an exercise intervention to increase exercise volume in a group of people with prodromal PD^75)^. This will have important implications for physiotherapy but also highlight opportunities for physiotherapists to engage further in exercise research in PD.

## Conclusion

Research over the last decade has strengthened the evidence for physiotherapy support of people with PD; expert neurologists now regard exercise as critical in symptom management^73)^. Further important questions remain; the intensity and optimal dose–response require further clarification, and strategies to best support long-term adherence, such as exergaming or remote coaching need further exploration. Further studies with early-stage cohorts will provide insights into whether the effects on motor function are symptomatic (delay disability progression) or are related to disease modification^5)^. Of equal importance, studies with people with mid-stage disease confirm the effectiveness of a range of modalities to delay the progression of hand, walking, and balance dysfunction, to reduce the risk of falling, and to support higher health-related quality of life.

The physiotherapy profession has an important challenge to support the goal that all people with PD can access evidence-based care as part of a multidisciplinary team across their lifespan. Expert skills in exercise prescription, and empowering chronic disease self-management are needed to tailor individual programs across the lifespan. Advocacy is needed to influence health policy to ensure equitable access and adequate funding. Education is needed at community and government levels to increase awareness of PD and the range of social supports needed. The engagement of physiotherapists in exercise-focused research is needed to ensure that the studies consider the implementation of the programs into the range of environments and contexts in which people with PD live.

## Funding

Not applicable.

## Conflicts of Interest

The authors have no conflicts of interest to declare.

## References

[1] World Health Organisation: Parkinson disease: A public health approach. Technical brief. Geneva; 2022

[2] Ministry of Health, Labor and Welfare. Japan intractable disease information center 2019. https://www.mhlw.go.jp/stf/english/index.html (Accessed April 28, 2024)

[3] Dorsey ER Elbaz A, *et al.*: GBD 2016 Parkinson’s Disease Collaborators: Global, regional, and national burden of Parkinson’s disease, 1990-2016: A systematic analysis for the Global Burden of Disease Study 2016. Lancet Neurol. 2018; 17: 939–953 30287051 10.1016/S1474-4422(18)30295-3 PMC6191528

[4] Chaudhuri KR Azulay JP, *et al.*: Economic burden of Parkinson’s disease: A multinational, real-world, cost-of-illness study. Drugs Real World Outcomes. 2024; 11: 1–11. 38193999 10.1007/s40801-023-00410-1 PMC10928026

[5] Bloem BR Okun MS, *et al.*: Parkinson’s disease. Lancet. 2021; 397: 2284–2303. 33848468 10.1016/S0140-6736(21)00218-X

[6] Camicioli R Morris ME, *et al.*: Prevention of falls in Parkinson’s disease: Guidelines and gaps. Mov Disord Clin Pract. 2023; 10: 1459–1469. 37868930 10.1002/mdc3.13860 PMC10585979

[7] Subramanian I Mathur S, *et al.*: Unmet needs of women living with Parkinson’s disease: Gaps and controversies. Mov Disord. 2022; 37: 444–455. 35060180 10.1002/mds.28921

[8] Goetz CG Poewe W, *et al.*: Movement disorder society task force report on the Hoehn and Yahr staging scale: Status and recommendations. Mov Disord. 2004; 19: 1020–1028. 15372591 10.1002/mds.20213

[9] Goldman JG Volpe D, *et al.*: Delivering multidisciplinary rehabilitation care in Parkinson’s disease: An international consensus statement. J Parkinsons Dis. 2024; 14: 135–166. 38277303 10.3233/JPD-230117 PMC10836578

[10] Grimes D Fitzpatrick M, *et al.*: Canadian guideline for Parkinson disease. CMAJ. 2019; 191: E989–E1004. 31501181 10.1503/cmaj.181504 PMC6733687

[11] Keus SH Munneke M, *et al.*: European physiotherapy guideline for Parkinson's disease. The Netherlands: KGNF/ParkinsonNet; 2014.

[12] National Institute of Health and Care Excellence: Parkinson’s disease in adults- NICE Guideline [NG71]. In: National Institute of Health and Care Excellence, editor. 2017.

[13] Osborne JA Botkin R, *et al.*: Physical therapist management of Parkinson disease: A clinical practice guideline from the American Physical Therapy Association. Phys Ther. 2022; 102: pzab302. 34963139 10.1093/ptj/pzab302 PMC9046970

[14] Development committee for Parkinson's disease treatment guideline. Parkinson’s Disease Clinical Practice Guidelines, Tokyo: Igaku Shoin; 2018. (in Japanese)

[15] Mizuno Y: Parkinsonian drugs: Guidelines for Japan. In: Riederer P Laux G et al. (eds): NeuroPsychopharmacotherapy, Springer, Cham, 2022, 3051–3078.

[16] Japanese Society of Physical Therapy: Neurological pathology therapy. Chap. 3. In: Guidelines for Physical Therapy. 2nd ed. 2020, 194–217. https://www.jspt.or.jp/guideline/2nd/ (in Japanese)

[17] Tomlinson CL Patel S, *et al.*: Physiotherapy versus placebo or no intervention in Parkinson’s disease. Cochrane Database Syst Rev. 2013; 9: CD002817. 10.1002/14651858.CD002817.pub4 PMC7120224 24018704

[18] Ernst M Folkerts AK, *et al.*: Physical exercise for people with Parkinson’s disease: A systematic review and network meta-analysis. Cochrane Database Syst Rev. 2023; 1: CD013856. 36602886 10.1002/14651858.CD013856.pub2 PMC9815433

[19] Gilat M Ginis P, *et al.*: A systematic review on exercise and training-based interventions for freezing of gait in Parkinson’s disease. NPJ Parkinsons Dis. 2021; 7: 81. 34508083 10.1038/s41531-021-00224-4 PMC8433229

[20] Proud EL Miller KJ, *et al.*: Effects of upper limb exercise or training on hand dexterity and function in people with Parkinson disease: A systematic review and meta-analysis. Arch Phys Med Rehabil. 2023. [Online ahead of print] 10.1016/j.apmr.2023.11.009 38042246

[21] Allen NE Canning CG, *et al.*: Interventions for preventing falls in Parkinson's disease. Cochrane Database of Syst Rev. 2022; 6: CD011574. 35665915 10.1002/14651858.CD011574.pub2 PMC9169540

[22] Tonkin PG Miller TD, *et al.*: The effects of exercise on non-motor experiences of daily living experienced in Parkinson’s disease: A systematic review and network meta-analysis. Clin Park Relat Disord. 2023; 9: 100203. 37293547 10.1016/j.prdoa.2023.100203 PMC10245098

[23] Yang Y Wang G, *et al.*: Efficacy and evaluation of therapeutic exercises on adults with Parkinson's disease: A systematic review and network meta-analysis. BMC Geriatr. 2022; 22: 813. 36271367 10.1186/s12877-022-03510-9 PMC9587576

[24] Hoffmann T Bakhit M, *et al.*: Shared decision making and physical therapy: What, when, how, and why? Braz J Phys Ther. 2022; 26: 100382. 35063699 10.1016/j.bjpt.2021.100382 PMC8784295

[25] Khan A Ezeugwa J, *et al.*: A systematic review of the associations between sedentary behavior, physical inactivity, and non-motor symptoms of Parkinson’s disease. PLoS One. 2024; 19: e0293382. 38551932 10.1371/journal.pone.0293382 PMC10980241

[26] Moratelli JA Lima AG, *et al.*: Evidence of physical activity interventions on non-motor symptoms of people with Parkinson’s disease: An umbrella review. Sport Sci Health. 2024. [Online ahead of print]

[27] Kim R Lee TL, *et al.*: Effects of physical exercise interventions on cognitive function in Parkinson’s disease: An updated systematic review and meta-analysis of randomized controlled trials. Parkinsonism Relat Disord. 2023; 117: 105908. 37922635 10.1016/j.parkreldis.2023.105908

[28] Costa V Prati JM, *et al.*: Physical exercise for treating the anxiety and depression symptoms of Parkinson’s disease: Systematic review and meta-analysis. J Geriatr Psychiatry Neurol. 2024. [Online ahead of print] 10.1177/08919887241237223 38445606

[29] Shulman LM Gruber-Baldini AL, *et al.*: The evolution of disability in Parkinson disease. Mov Disord. 2008; 23: 790–796. 18361474 10.1002/mds.21879

[30] Maetzler W Mirelman A, *et al.*: Identifying subtle motor deficits before Parkinson’s disease is diagnosed: What to look for? J Parkinsons Dis. 2024. [Online ahead of print] 10.3233/JPD-230350 PMC11492040 38363620

[31] Ellis TD Colon-Semenza C, *et al.*: Evidence for early and regular physical therapy and exercise in Parkinson’s disease. Semin Neurol. 2021; 41: 189–205. 33742432 10.1055/s-0041-1725133 PMC8678920

[32] Collett J Brusco N, *et al.*: Lost employment potential and supporting people with Parkinson’s to stay in work: Insights from a Pan European cross-sectional survey. Disabil Rehabil. 2023; 45: 832–839. 35249423 10.1080/09638288.2022.2043460

[33] Lord S Godfrey A, *et al.*: Ambulatory activity in incident Parkinson’s: More than meets the eye? J Neurol. 2013; 260: 2964–2972. 23900754 10.1007/s00415-013-7037-5

[34] Lord S Galna B, *et al.*: Natural history of falls in an incident cohort of Parkinson’s disease: Early evolution, risk and protective features. J Neurol. 2017; 264: 2268–2276. 28948348 10.1007/s00415-017-8620-y PMC5656700

[35] Ellis T Rochester L: Mobilizing Parkinson’s disease: The future of exercise. J Parkinsons Dis. 2018; 8(s1): S95–S100. 30584167 10.3233/JPD-181489 PMC6311359

[36] Frazzitta G Maestri R, *et al.*: Intensive rehabilitation treatment in early Parkinson’s disease: A randomized pilot study with a 2-year follow-up. Neurorehabil Neural Repair. 2015; 29: 123–131. 25038064 10.1177/1545968314542981

[37] Schenkman M Moore CG, *et al.*: Effect of high-intensity treadmill exercise on motor symptoms in patients with de novo Parkinson disease: A phase 2 randomized clinical trial. JAMA Neurol. 2018; 75: 219–226. 29228079 10.1001/jamaneurol.2017.3517 PMC5838616

[38] Handlery R Stewart JC, *et al.*: Physical activity in de novo Parkinson disease: Daily step recommendation and effects of treadmill exercise on physical activity. Phys Ther. 2021; 101: pzab174. 34244805 10.1093/ptj/pzab174

[39] Li JA Loevaas MB, *et al.*: Does exercise attenuate disease progression in people with Parkinson’s disease? A systematic review with meta-analyses. Neurorehabil Neural Repair. 2023; 37: 328–352. 37166181 10.1177/15459683231172752 PMC10272626

[40] Tsukita K Sakamaki-Tsukita H, *et al.*: Long-term effect of regular physical activity and exercise habits in patients with early Parkinson disease. Neurology. 2022; 98: e859–e871. 35022304 10.1212/WNL.0000000000013218 PMC8883509

[41] Miller SA Mayol M, *et al.*: Rate of progression in activity and participation outcomes in exercisers with Parkinson’s disease: A five-year prospective longitudinal study. Parkinsons Dis. 2019; 2019: 5679187. 31662843 10.1155/2019/5679187 PMC6778930

[42] Rafferty MR MacDonald J, *et al.*: Using implementation frameworks to provide proactive physical therapy for people with Parkinson disease: Case report. Phys Ther. 2019; 99: 1644–1655. 31508801 10.1093/ptj/pzz129 PMC7372734

[43] Ellis T Cavanaugh JT, *et al.*: Factors associated with exercise behavior in people with Parkinson disease. Phys Ther. 2011; 91: 1838–1848. 22003171 10.2522/ptj.20100390 PMC3229047

[44] Ellis T Boudreau JK, *et al.*: Barriers to exercise in people with Parkinson disease. Phys Ther. 2013; 93: 628–636. 23288910 10.2522/ptj.20120279 PMC3641403

[45] Zaman A Ellingson L, *et al.*: Determinants of exercise behaviour in persons with Parkinson’s disease. Disabil Rehabil. 2021; 43: 696–702. 31322434 10.1080/09638288.2019.1638975

[46] Mc Ardle R Del Din S, *et al.*: Factors influencing habitual physical activity in Parkinson’s disease: Considering the psychosocial state and wellbeing of people with Parkinson’s and their carers. Sensors (Basel). 2022; 22: 871. 35161617 10.3390/s22030871 PMC8837970

[47] van Nimwegen M Speelman AD, *et al.*: Promotion of physical activity and fitness in sedentary patients with Parkinson’s disease: Randomised controlled trial. BMJ. 2013; 346(mar01 2): f576. 23457213 10.1136/bmj.f576 PMC3585777

[48] Lai B Bond K, *et al.*: Exploring the uptake and implementation of tele-monitored home-exercise programmes in adults with Parkinson’s disease: A mixed-methods pilot study. J Telemed Telecare. 2020; 26: 53–63. 30134777 10.1177/1357633X18794315

[49] Colón-Semenza C Latham NK, *et al.*: Peer coaching through mhealth targeting physical activity in people with Parkinson disease: Feasibility study. JMIR Mhealth Uhealth. 2018; 6: e42. 29449201 10.2196/mhealth.8074 PMC5832905

[50] Ellis T Latham NK, *et al.*: Feasibility of a virtual exercise coach to promote walking in community-dwelling persons with Parkinson disease. Am J Phys Med Rehabil. 2013; 92: 472–481, quiz 482-485. 23552335 10.1097/PHM.0b013e31828cd466 PMC4266140

[51] Ellis TD Cavanaugh JT, *et al.*: Comparative effectiveness of mhealth-supported exercise compared with exercise alone for people with Parkinson disease: Randomized controlled pilot study. Phys Ther. 2019; 99: 203–216. 30715489 10.1093/ptj/pzy131

[52] Hermanns M Haas BK, *et al.*: Engaging older adults with Parkinson’s disease in physical activity using technology: A feasibility study. Gerontol Geriatr Med. 2019; 5: 2333721419842671. 31069250 10.1177/2333721419842671 PMC6492351

[53] Ahern L Timmons S, *et al.*: A systematic review of Behaviour Change Interventions to improve exercise self-efficacy and adherence in people with Parkinson’s disease using the Theoretical Domains Framework. J Frailty Sarcopenia Falls. 2024; 9: 66–88. 38444546 10.22540/JFSF-09-066 PMC10910257

[54] Alberts JL Rosenfeldt AB, *et al.*: Effectiveness of a long-term, home-based aerobic exercise intervention on slowing the progression of Parkinson disease: Design of the cyclical lower extremity exercise for Parkinson disease II (CYCLE-II) study. Phys Ther. 2021; 101: pzab191. 34363478 10.1093/ptj/pzab191 PMC8632855

[55] Mirelman A Rochester L, *et al.*: Addition of a non-immersive virtual reality component to treadmill training to reduce fall risk in older adults (V-TIME): A randomised controlled trial. Lancet. 2016; 388: 1170–1182. 27524393 10.1016/S0140-6736(16)31325-3

[56] Pahwa R Isaacson SH, *et al.*: Role of the Personal KinetiGraph in the routine clinical assessment of Parkinson’s disease: Recommendations from an expert panel. Expert Rev Neurother. 2018; 18: 669–680. 30032695 10.1080/14737175.2018.1503948

[57] Kehagia AA Chowienczyk S, *et al.*: Real-world evaluation of the feasibility, acceptability and safety of a remote, self-management Parkinson’s disease care pathway: A healthcare improvement initiative. J Parkinsons Dis. 2024; 14: 197–208. 38250784 10.3233/JPD-230205 PMC10836560

[58] McDermott A Bradley D. Physiotherapists’ perceptions of the use of digital health to promote physical activity in people living with Parkinson’s disease. Euro J Physiotherapy. 2023. [Online ahead of print]

[59] Omberg L Chaibub Neto E, *et al.*: Remote smartphone monitoring of Parkinson’s disease and individual response to therapy. Nat Biotechnol. 2022; 40: 480–487. 34373643 10.1038/s41587-021-00974-9 PMC12812035

[60] Schootemeijer S de Vries NM, *et al.*: The STEPWISE study: study protocol for a smartphone-based exercise solution for people with Parkinson’s Disease (randomized controlled trial). BMC Neurol. 2023; 23: 323. 37700241 10.1186/s12883-023-03355-8 PMC10496249

[61] Kwon SH Park JK, *et al.*: A systematic review and meta-analysis on the effect of virtual reality-based rehabilitation for people with Parkinson’s disease. J Neuroeng Rehabil. 2023; 20: 94. 37475014 10.1186/s12984-023-01219-3 PMC10360300

[62] Marotta N Demeco A, *et al.*: Nintendo Wii(TM) versus Xbox Kinect(TM) for functional locomotion in people with Parkinson’s disease: A systematic review and network meta-analysis. Disabil Rehabil. 2022; 44: 331–336. 32478581 10.1080/09638288.2020.1768301

[63] Darweesh SKL Sampaio C, *et al.*: Has the time come to redefine Parkinson’s disease? Lancet Neurol. 2024; 23: 130–133. 38267174 10.1016/S1474-4422(23)00503-3

[64] Rizzo G Copetti M, *et al.*: Accuracy of clinical diagnosis of Parkinson disease: A systematic review and meta-analysis. Neurology. 2016; 86: 566–576. 26764028 10.1212/WNL.0000000000002350

[65] Siderowf A Concha-Marambio L, *et al.*: Assessment of heterogeneity among participants in the Parkinson’s Progression Markers Initiative cohort using α-synuclein seed amplification: A cross-sectional study. Lancet Neurol. 2023; 22: 407–417. 37059509 10.1016/S1474-4422(23)00109-6 PMC10627170

[66] Schalkamp AK Peall KJ, *et al.*: Wearable movement-tracking data identify Parkinson’s disease years before clinical diagnosis. Nat Med. 2023; 29: 2048–2056. 37400639 10.1038/s41591-023-02440-2

[67] van der Kolk NM de Vries NM, *et al.*: Effectiveness of home-based and remotely supervised aerobic exercise in Parkinson’s disease: A double-blind, randomised controlled trial. Lancet Neurol. 2019; 18: 998–1008. 31521532 10.1016/S1474-4422(19)30285-6

[68] Alberts JL Rosenfeldt AB: The universal prescription for Parkinson’s disease: Exercise. J Parkinsons Dis. 2020; 10(s1): S21–S27. 32925109 10.3233/JPD-202100 PMC7592674

[69] Patterson CG Joslin E, *et al.*: Study in Parkinson’s disease of exercise phase 3 (SPARX3): Study protocol for a randomized controlled trial. Trials. 2022; 23: 855. 36203214 10.1186/s13063-022-06703-0 PMC9535216

[70] de Laat B Hoye J, *et al.*: Intense exercise increases dopamine transporter and neuromelanin concentrations in the substantia nigra in Parkinson’s disease. NPJ Parkinsons Dis. 2024; 10: 34. 38336768 10.1038/s41531-024-00641-1 PMC10858031

[71] Johansson ME Cameron IGM, *et al.*: Aerobic exercise alters brain function and structure in Parkinson’s disease: A randomized controlled trial. Ann Neurol. 2022; 91: 203–216. 34951063 10.1002/ana.26291 PMC9306840

[72] Fang X Han D, *et al.*: Association of levels of physical activity with risk of Parkinson disease: A systematic review and meta-analysis. JAMA Netw Open. 2018; 1(5): e182421. 30646166 10.1001/jamanetworkopen.2018.2421 PMC6324511

[73] Janssen Daalen JM Schootemeijer S, *et al.*: Lifestyle interventions for the prevention of Parkinson disease: A recipe for action. Neurology. 2022; 99(Suppl. 1): 42–51. 10.1212/WNL.0000000000200787 35970584

[74] Oosterhof TH Schootemeijer S, *et al.*: Clinical trial highlights - Interventions promoting physical activity in Parkinson’s disease. J Parkinsons Dis. 2023; 13: 311–322. 37125564 10.3233/JPD-239001 PMC10200135

[75] Darweesh SKL Bloem BR: Using integrated biomarkers of prodromal Parkinson’s disease to assess the efficacy of exercise intervention in the earliest phase of disease. Michael J Fox Foundation. 2022

